# Cariogenic Risk and COVID-19 Lockdown in a Paediatric Population

**DOI:** 10.3390/ijerph18147558

**Published:** 2021-07-15

**Authors:** Raffaella Docimo, Micaela Costacurta, Paola Gualtieri, Alberto Pujia, Claudia Leggeri, Alda Attinà, Giulia Cinelli, Silvia Giannattasio, Tiziana Rampello, Laura Di Renzo

**Affiliations:** 1 Pediatric Dentistry, Department of Surgical Sciences, University of Rome Tor Vergata, 00133 Rome, Italy; raffaelladocimo@tiscali.it (R.D.); micaela.costacurta@uniroma2.it (M.C.); 2 Section of Clinical Nutrition and Nutrigenomics, Department of Biomedicine and Prevention, University of Rome Tor Vergata, Via Montpellier 1, 00133 Rome, Italy; laura.di.renzo@uniroma2.it; 3 Department of Biomedicine and Prevention, University of Rome Tor Vergata, Via Montpellier 1, 00133 Rome, Italy; albpujia@gmail.com; 4 Department of Biomedicine and Prevention, School of Specialization in Food Sciences, University of Rome Tor Vergata, Via Montpellier 1, 00133 Rome, Italy; claudialeggeri@gmail.com (C.L.); alda.attina@gmail.com (A.A.); silviagiannattasio85@gmail.com (S.G.); tizianarampello1@gmail.com (T.R.); 5 Predictive and Preventive Medicine Research Unit, Bambino Gesù Children Hospital IRCCS, 00165 Rome, Italy; giulia.cinelli@opbg.net

**Keywords:** dental caries, eating habits, lifestyle, COVID-19 pandemic

## Abstract

The Severe Acute Respiratory Syndrome Coronavirus 2 disease COVID-19 pandemic caused several lifestyle changes, especially among younger people. The study aimed to describe the impact of eating habits, lifestyle, and home oral hygiene during the COVID-19 pandemic, on the cariogenic risk in the Italian paediatric population, by using an online survey. The survey was conducted through a virtual questionnaire divided into four parts: child personal and anthropometric data; oral health; child dietary habits (KIDMED test); and child lifestyle, before and during COVID-19 lockdown. During the lockdown, only 18.6% of the participants had high adherence to a Mediterranean diet, recording an increase in sweets consumption and the number of meals (*p* < 0.001). In terms of lifestyle, the percentage of moderately and vigorously active children decreased (41.4% and 5.0%, respectively) (*p* = 0.014). The percentage of children sleeping more than 9 h increased (*p* < 0.001). They watched more television programs (*p* < 0.001). Regarding oral hygiene, children did not change their brushing habits (*p* = 0.225). The percentage of children using non-fluoridated toothpaste was higher (6.4%), and no changes were observed (*p* > 0.05). In some cases, dental pain and abscesses were declared (10% and 2.7%, respectively). This study confirms the need for campaigns to promote hygiene and dental care in combination with food education for a correct habit and promotion of a healthy and sustainable dietary style.

## 1. Introduction

In the last year, our world has changed dramatically due to a new pandemic virus that spread all over the globe: Severe Acute Respiratory Syndrome Coronavirus 2 (SARS-CoV-2), a new form of coronavirus that has triggered a worldwide state of emergency, causing a strong impact on our lifestyles and eating habits. The lockdown has led to a rapid change in the habits and lifestyles of the population: physical distancing, reduction of socialization and relationship life, increase in time spent at home, digital education, smart working, and limitation of physical activity outdoors and in closed places (gyms, swimming pools).

Since March 2020, several studies have been conducted to understand almost all of the changes in everyday life in different population categories and under precise circumstances [1].

According to an Italian survey conducted on 3533 subjects between the ages of 12 and 86, changes in habits and lifestyles during the lockdown influenced eating habits and adherence to the Mediterranean diet model, particularly in the younger and older populations [2].

Regarding Italy, the country has been subjected to several restriction laws that characterized different periods as part of a “hard” lockdown (from March to May 2020). During the tougher times, all non-necessary shops were closed, going out was banned, and people were encouraged to work online. At other times, people had the chance to go out and go to work, and almost all the shops had reopened [3]. In all these periods, people had to wear masks, maintain a distance of at least 1 m, and wash their hands frequently.

The lockdown has caused several health and psychological problems in different subjects, including young people and children [2]. Changes in eating habits occurred during the hard lockdown, and in some cases, they continued to persist in the “soft” days. In particular, people had demonstrated to have depressed moods, anxious feelings, and increased food intake, preferring comfort food in particular [2]. Furthermore, the lockdown period caused several emotional problems in the Italian population and in their eating habits, because people tended to eat foods rich in sugars and with a high caloric content [4].

This new situation compromised the maintenance of a healthy and varied diet and introduced incorrect habits that could increase cariogenic risk.

Dental caries is one of the most common childhood diseases, and it continues into adulthood [5]. Globally, 2.3 billion people suffer from caries of permanent teeth, and more than 530 million children suffer from caries of primary teeth [6].

The risk factors for pathological caries are frequently caused by the consumption of dietary sugars, inadequate fluoride, poor oral hygiene, and salivary dysfunction, whereas the protective factors are a healthy diet, brushing with fluoride toothpaste twice a day, professional topical fluoride, preventive and therapeutic sealants, and normal salivary function [7].

Therefore, caries risk assessment is complex because a variety of social, cultural, behavioural, and socio-economic factors [8].

In a study conducted in Southern Italy on a sample of non-migrant, economically disadvantaged children (age range: 12–14 years old), a Decayed, Missing, and Filled Teeth (DMFT) index of 3.29 ± 3.21 and caries prevalence of 55.9% were highlighted [9].

Dental caries is a biofilm-mediated, sugar-driven, multifactorial, dynamic disease that is determined by the loss of the mineral homeostasis of tooth surfaces and by the imbalance between the demineralization and remineralization phases of the enamel. The pathological caries risk factors shift the balance in the direction of demineralization, dental caries, and lesion progression, whereas protective factors promote remineralization, lesion arrest, or regression [7].

Although dental caries has a microbial aetiology, dietary sugar has been the most important risk factor [10].

Healthy eating behaviours appear already in early childhood; in fact, the first months of life are crucial for the learning process of taste in humans and the subsequent acceptance of foods, especially healthy ones [11]. A diet rich in sugars from early childhood is one of the risk factors in determining the onset of early childhood caries (ECC) [12,13].

The total energy intake is the sum of all daily calories/kilojoules introduced with the diet, including macronutrients, such as fat, protein, carbohydrate-included total sugars (free sugars + intrinsic sugars + milk sugars), dietary fibers, and ethanol (i.e., alcohol). Free sugars include monosaccharides and disaccharides added to foods and beverages by the manufacturer, cook, or consumer; and sugars naturally present in honey, syrups, fruit juices, and fruit-juice concentrates [14].

The World Health Organization (WHO, Geneva, Switzerland) recommends a reduced intake of free sugars throughout the life course (strong recommendation), with a reduction of free-sugar intake to less than 10% of the total energy intake (strong recommendation), and preferably below 5% of the total energy intake (conditional recommendation) in both adults and children [14].

According to a systematic review of the literature to inform WHO guidelines, it appears that 42 out of 50 studies conducted on children reported a positive association between sugar intake and caries. According to the authors, there was moderate-quality evidence showing that caries is lower when free-sugar intake is <10% of the total energy intake. With a <5% energy cut-off, the risk of caries could be further reduced, but the evidence was judged to be not strong enough [10].

The importance of the WHO Sugar Guidelines for Dental Health and Obesity Prevention was also shared in the joint European Organisation for Caries Research (ORCA)/ European Association of Dental Public Health (EADPH) symposium on sugar and oral health in 2016 [15].

Studies in the literature often evaluated the frequency of intake of added sugars, rather than free sugars and their association with caries pathology in pediatric age [16]. Added sugars are more restrictively defined than free sugars, being monosaccharides and disaccharides added to foods and beverages by the cook or consumer. Hence, the level of free-sugar intake is probably even higher than that reported in most national data [15].

A review of sugar consumption from national representative dietary surveys across the world reported that intakes of added sugars were higher in school-aged children and adolescents (up to 19% of total energy) compared to younger children or adults [17]. In Europe, added sugars contributed 11–17% of total energy intake in children, and were provided mostly by sweet products (40 to 50%) (e.g., confectionery, chocolates, cakes, biscuits, sugar, and jam), beverages (20 to 34%) (e.g., sugar-sweetened beverages and fruit nectars, excluding fruit juices), dairy products (6 to 18%) (e.g., yogurt, milk-based desserts) [18].

The purpose of this study was to analyze, in a pediatric population, the cariogenic risk related to changes in eating habits, lifestyle, and home oral hygiene during the COVID-19 lockdown.

## 2. Materials and Methods

### 2.1. Survey Methodology

The Eating Habits and Lifestyle Changes in COVID-19 lockdown (EHLC-COVID-19) project [2] is directed by the Section of Clinical Nutrition and Nutrigenomics, Department of Biomedicine and Prevention of the University of Rome Tor Vergata. The third part of the EHLC-COVID-19 project investigates the cariogenic risk in childhood. It was made by the Paediatric Dentistry Post-Graduate School of the University of Rome Tor Vergata. For this occasion, a web survey (Table A1) was launched to obtain data on changes in dietary lifestyle, oral hygiene, and oral health of the Italian paediatric population, from 4 to 14 years old.

The survey was conducted for 6 months, from 15 July 2020 to 15 January 2021, among the Italian paediatric population by using an online platform accessible with an Internet connection through any device. The survey was diffused through institutional and private social networks (Twitter, Facebook, and Instagram), the “PATTO in Cucina Magazine” website [19], and institutional mailing lists. The survey was completed by one of the parents. This administrative methodology provided a statistical collective sample, hence the population parameters could not be controlled, as is the case for probabilistic sampling. Nevertheless, it was completely efficacious for the research objective, since it facilitated the wide dissemination of the survey throughout Italian regions during a time when, due to the pandemic, there were many territorial restrictions. Furthermore, the annual Italian report on the use of the Internet reported that Internet penetration stood at 82% in January 2020; in particular, 94% of internet users, from 16 to 64 years old, used their smartphone to connect, and 99% of them visited or used a messaging service or a social network [20].

The study was carried out in full agreement with national and international regulations, and the Declaration of Helsinki (2000). Parents were fully informed about the study requirements and were asked to accept the data-sharing and privacy policy before filling out the questionnaire, which was directly connected to the Google Form platform. The parents’ and child’s personal information, including names, were anonymized to maintain and protect confidentiality. The web survey was anonymous and did not allow us to trace sensitive personal data in any way. Moreover, the present web survey study did not need approval from the Ethics Committee. Once completed, each response was transmitted to the Google platform and the final database was downloaded as a Microsoft Excel sheet.

### 2.2. Mediterranean Diet Quality Index (KIDMED) Questionnaire

The levels of adherence to the Mediterranean diet (MD) in our study were measured with the KIDMED index.

The KIDMED test is the most used test in the literature to assess adherence to the MD in children and young people. KIDMED was devised by Serra-Majem et al. in subjects between the ages of 2 and 24, focusing on specific eating habits of the MD. The questionnaire could be self-administered or completed by interview (paediatrician, dietician, etc.).

The 16 KIDMED questions ranged from 0 to 12 points. Most of the questions concerned the frequency of consumption of different food groups and their portions without specifying grams. The score was assigned as follows: positive adherence to the MD, +1; and negative adherence, −1. The sum of the values from the tests was divided into three levels: >8, excellent adherence to the MD; 4–7, need to improve daily intake according to MD models; ≤3, non-congruent quality with the MD model [21].

### 2.3. Cariogenic Risk during COVID-19 Questionnaire

The questionnaire on cariogenic risk during COVID-19 was specifically structured using Google Forms by the Section of Clinical Nutrition and Nutrigenomics, Department of Biomedicine and Prevention, of the University of Rome Tor Vergata, and by the Paediatric Dentistry Post-Graduate School of the University of Rome Tor Vergata. The questionnaire included 56 questions divided into 4 different sections: (1) child’s personal and anthropometric data (7 questions: gender, age, region and province of residence, height, weight, and type of breastfeeding in the first 6 months of life); (2) oral health before and during the COVID-19 lockdown (8 questions: cariogenic status, dental pain, presence of dental abscesses, number of brushings per day, type of toothpaste, brushing habits); (3) child dietary-habit information before and during the COVID-19 lockdown: (a) 8 questions (number of meals/day; out of meals/day, frequency of consumption of sweetened foods; probiotic use), (b) 16 questions: adherence to the MD among children and adolescence (KIDMED) [21], (c) 4 questions: sweet snacks per day, eggs per day, daily water consumption; and (4) child’s lifestyle before and during the COVID-19 lockdown (13 questions: sports habits, quality of sleep, hours of TV per day, hours of distance learning). Specific questions about physical-activity habits were modified from a survey conducted by Istituto Superiore di Sanità [22]. The full version of the questionnaire is presented in Appendix A. Based on the KIDMED index, participants were divided into 3 classes: (1) poor adherence (score ≤3 points), (2) medium adherence (score 4–7 points), and (3) high adherence (score ≥8) to the MD, and differences in the compliance rates for each food were calculated.

### 2.4. Statistical Analyses

Data are represented as numbers and percentages in parentheses (%) for categorical variables or mean and standard deviation (SD) for continuous variables. The Shapiro–Wilk test was performed to evaluate the distribution of variables. All the variables had a skewed distribution. The Spearman correlation coefficient was calculated to evaluate the correlation between continuous variables. A chi-squared test was employed to assess the association between categorical variables, while McNemar’s analysis was used to investigate the difference between categorical variables before and during the COVID-19 emergency. Mann–Whitney U and Kruskal–Wallis tests were performed to compare continuous variables among two or more groups, respectively. Finally, binary and multinomial logistic regression analyses were conducted to investigate the association between categorical variables (dependent) and continuous or categorical ones (independent). Results were significant for *p*-value < 0.05. Statistical analysis was performed using SPSS ver. 21.0 (IBM, Chicago, IL, USA).

## 3. Results

### 3.1. Participants

**Figure A1:**
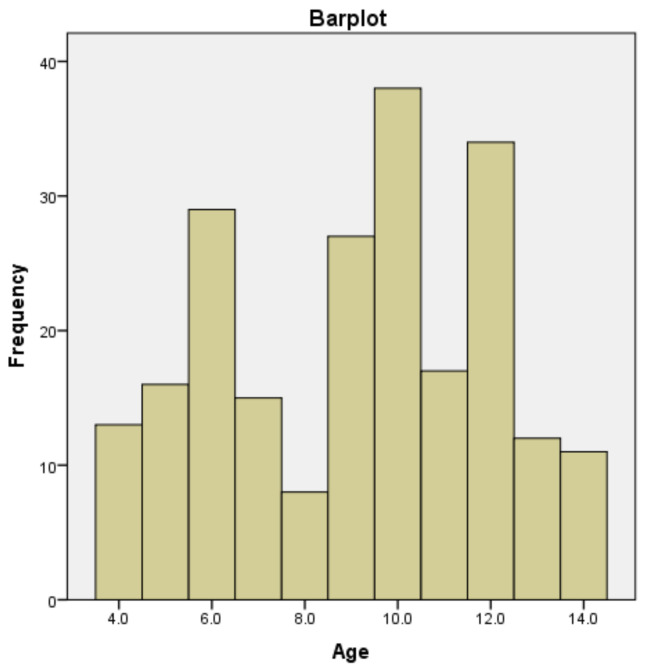
Age distribution of the sample (*n* = 220). Non-normal distribution; Shapiro–Wilk test: *p* < 0.001.

On 15 January 2021, the web survey was concluded and the collected data were analyzed. A total of 225 participants completed the questionnaire. Five interviewees did not give their consent for the data treatment; hence, 220 subjects, aged between 4 and 14 years old, were included in the data analysis. The age distribution of the sample is shown in Figure A1. The sample population was homogeneous by gender, in particular with females representing 50.5% of the sample. General characteristics and anthropometrics of the population are reported in Table 1.

The territorial coverage extended to the following Italian regions: Lazio (76.4%), Abruzzo (4.1%), Lombardy (2.7%), Calabria (5.9%), Apulia (4.5%), Emilia Romagna (0.9%), Veneto (1.4%), Campania (1.4%), Tuscany (0.5%), and Molise (2.3%). The majority of the interviewees lived in Rome (57.7%).

To evaluate compliance with the MD recommendations during the COVID-19 lockdown, the KIDMED questionnaire was included in the survey. Table 2 shows the percentage of positive answers to each question. The table also includes the percentage of positive answers concerning changes in eating habits and increases in consumption of sweets. In particular, data showed a great consumption of some MD-typical foods, such as extra virgin olive oil (99.5%) and cereals (89.5%). Conversely, only half of the participants had a good intake of legumes (50.5%) and fish (50.0%), while only one out of three children consumed two portions of fruits (32.7%) or vegetables (28.6%) per day. Finally, concerning poor eating habits, the KIDMED test showed that three and one out of 4 kids, respectively, consumed biscuits, cookies, and snacks (75%) or skipped breakfast (25.5%) during the lockdown.

The KIDMED score showed that only 18.6% of the participants had high adherence to the MD during the lockdown (Table 2).

Regarding daily water consumption during the lockdown period, 46.4% of children drank between 1 and 2 L of water per day according to the reference intake levels for the Italian population [23]. (Table 2).

Concerning eating habits, half of the participants had not changed their eating habits (50.9%), but the data showed an increase in sweets consumption during the COVID-19 lockdown (Table 2). Before and during the COVID-19 lockdown, the percentage of participants who ate outside of meals increased from 25.9% to 54.5% after the lockdown (*p* < 0.001). The number of meals before and during the lockdown also increased (*p* < 0.001) (before: 4.16 ± 0.96; during: 4.45 ± 1.19). No difference between the two periods was found concerning the use of probiotic supplements (*p* = 0.475) (14.5% before vs. 12.3% during).

### 3.2. Lifestyle

**Figure 1:**
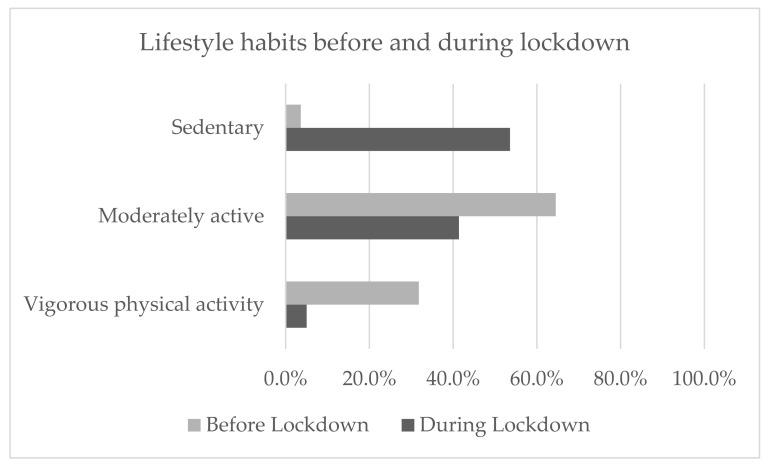
Lifestyle habits before and during the lockdown split by sedentary, moderately active, and vigorous physical activity. A chi-squares analysis was performed to compare lifestyle habits before and during the lockdown (*p* < 0.001).

Lifestyle habits before and during lockdown changed significantly (*p* < 0.001). 53.6% of the whole sample declared to have followed a more sedentary lifestyle during the lockdown, compared to the 3.6% who were sedentary before; the percentage of moderately active children decreased from 64.5% to 41.4%; while the percentage of children who had vigorous physical activity before the lockdown changed from 31.8% to 5.0% (Figure 1).

**Figure 2:**
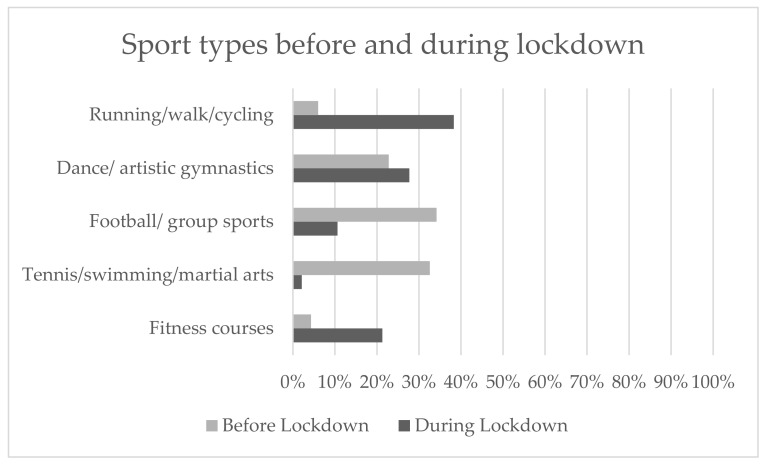
Percentages of the whole sample sport types before and during the lockdown. The McNemar’s test was performed to compare different sports before and during the lockdown (*p* = 0.014).

With regard to sports, children had stopped or limited some sports that required specific gym areas or less social distancing; e.g., tennis/swimming/martial arts, football/group courses, dance/artistic gymnastics, and others. A statistically significant difference before and during lockdown was found (*p* = 0.014) (Figure 2).

**Figure 3:**
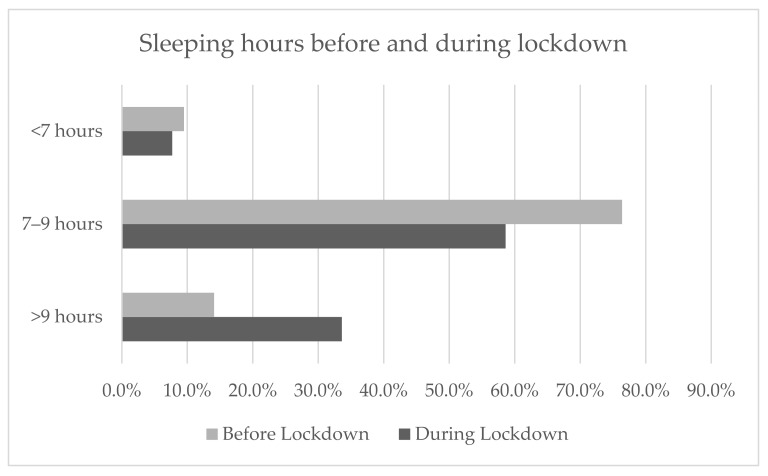
Percentages of the whole sample sleeping hours before and during the lockdown. The McNemar’s test was performed to compare sleeping hours before and during the lockdown (*p* < 0.001).

With regard to changes in sleeping hours, the statistical analysis showed that during the lockdown, children slept more than they did before. In particular, the percentage of children sleeping more than 9 h increased from 14.1% to 33.6%. A statistically significant difference was found (*p* < 0.001) (Figure 3).

Children also watched more television programs (TV) during the lockdown compared to before (*p* < 0.001) (before: 1.98 ± 1.13 h; during: 3.66 ± 4.00 h).

### 3.3. Oral Hygiene

Regarding oral hygiene in children before and during the lockdown, there was no difference in fluoride toothpaste usage, with 55.0% and 33.6% of children often or always using it regardless (*p* = 0.225). Results also showed that children had not changed their sleeping hygiene routines and continued to brush their teeth before going to sleep (*p* = 0.338). Conversely, there was a significant difference in terms of drinking or eating after tooth brushing, showing that children had increased this habit during lockdown (Table 3).

Furthermore, during the lockdown, some of the interviewees declared dental pain and abscesses (10% and 2.7%, respectively). However, they had not changed their brushing habits, and they used their personal toothpaste (75% and 66.4%, respectively) (Table 3).

Some of the subjects did not treat their caries during lockdown (7.3%).

## 4. Discussion

The results analysis showed that the COVID-19 lockdown led to substantial changes in the dietary and eating habits of families. Although the survey was disseminated on social networks or mailing lists and therefore covered the entire Italian territory, the majority of respondees (76.4%) were from Lazio. It is probable that parents belonging to our department or university responded more. However, being an anonymous survey, this remains a supposition.

The KIDMED questionnaire results showed that most children’s diets during the pandemic were normally adherent to the MD model. According to MD adherence, children with a low, medium, or high score had an adequate consumption of extra virgin olive oil, fish, legumes, milk, yogurt, pasta, and rice. Following our results, some studies reported that dietary behaviours improved (e.g., daily fruit intake), while others worsened (e.g., sweetened-beverage intake) in Italian children during the COVID-19 lockdown [24]. According to Medrano et al., the improvement of habits during the pandemic may have been due to an increase in available time and interest in cooking [25]. On the other hand, the KIDMED test showed an increase in the consumption of foods far from the MD regime, characterized by the consumption of plant-based and fish-based foods, and reduced consumption of meat and dairy products except for milk, yogurt, and seasoned cheese [26].

Our results showed that during the lockdown, there was an increase in the consumption of sweet products, biscuits, and snacks, and reduced consumption of foods such as fruit, vegetables, and nuts. The consumption of sweets increased in 51.4% of the sample, with an additional change in the number of meals (4.16% before lockdown, 4.45% during lockdown; *p* < 0.001), and in eating behaviours between meals (25.4% before lockdown, 54.5% during lockdown; *p* < 0.001).

Similar results were found in a recent study by López-Bueno et al., who observed a significant reduction of fruit and vegetable consumption in children aged 3 to 5 during the COVID-19 lockdown [27]. Similarly, a study focused on Italian teenagers and children reported an increase in the consumption of red meat, chips, and sweetened beverages during the COVID-19 lockdown [24]. A possible explanation for these findings could be due to parents’ difficulty in preparing complete and healthy meals for their children, as they had to reconcile smart working with family life. This led to a worsening in eating habits, especially for younger and less-independent children, because they could not cook for themselves [27].

Many studies on children’s sedentary behaviour have been published recently: e.g., on changes in physical activity level (PAL) in American children, which led to an increased risk of obesity, diabetes, and cardiovascular diseases [28]. These drastic changes were strictly connected with sleep and quality regression in both children and adolescents [29]. It was also noted that a low PAL interacted with body fat and appetite dysregulation. Deleterious effects have been described after a sudden interruption of PA, as it has been linked to insulin resistance in muscle tissue and decreased muscle glucose utilization and atrophy. Cardiovascular benefits were lost after two weeks of inactivity, with augmented production of atherogenic lipoproteins and obesity promotion [30].

In our results for Italian children, we observed that our interviewees changed their lifestyle habits during the lockdown, preferring a more sedentary behaviour compared to before. This aspect influenced sport decisions, because they suddenly stopped their workouts, preferring individual open-air sports (i.e., running, walking, and cycling). Changes in sports activity and lifestyle influenced sleeping quality and routines. Children tended to sleep more during lockdown compared to before. These data probably are connected to an increase in hours spent watching TV. Our data showed an increase in hours/day spent watching TV (2.00 before lockdown, 4.00 during lockdown; *p* < 0.001), which could be related to the increase in the frequency of eating between meals. According to a recent study, children aged 10–12 who watched TV (>90 min per day) were more likely to consume cariogenic foods and develop caries disease [31].

All these parameters led to the conclusion that the lockdown negatively influenced children’s health and routines. In these situations, parents’ moods are crucial. McCormack et al. highlighted that during the lockdown, more-anxious parents tended to let their children stay at home watching TV or playing computer games than less-anxious parents. Hence, parents also had a strong impact on the lifestyle habits of their children [32].

The COVID-19 lockdown may harm children’s mental health and potentially promote monotony, distress, impatience, annoyance, and varied neuropsychiatric manifestations [33]. These psychosocial aspects can generate overeating and an increase in “comfort food” consumption, defined as “food craving”. According to an Italian survey during the COVID-19 lockdown, the “comfort food” was rich in sugar (42.5%), notably chocolate, ice-cream, desserts, and salty snacks (23.5%) [34]. These foods, mainly rich in simple carbohydrates, can reduce stress, as they promote the production of serotonin, which affects the mood positively [35].

According to a study conducted in France on parents of 498 children aged 3–12 during the COVID-19 lockdown, increased child boredom significantly predicted increased food responsiveness, emotional overeating, and snack eating between meals. Parental behaviour had also changed (more permissive, fewer rules, more soothing with food, more child autonomy) [36].

Jansen et al. showed an association between parental stress in the period of the COVID-19 lockdown, with children given foods particularly rich in kcals during snack times and more processed snacks with low nutrients (i.e., emotional and instrumental feeding) [37].

The limited possibilities for daily shopping during the COVID-19 lockdown also led to changes in food choice that may have reduced the consumption of fresh foods (e.g., fruit, vegetables, and fish) in favour of highly processed ones (e.g., cheap foods, junk foods, snacks) rich in sugar, fats, and salt. These sugar-rich dietary habits and the intake of energy-dense, low-nutrient-dense snack foods may increase cariogenic risk.

The intake of beverages and foods containing simple carbohydrates is not recommended between meals [38], and several studies asserted that snack intake between meals, carried out one or more times a day, increased the risk of dental caries [39]. The excess energy intake associated with free sugars can determine dental caries, as well as excess body weight, leading to an increased likelihood of overweight and obesity [14]. There is a strong relationship between prevalence of dental caries and body-fat percentage measured by DXA (dual X-ray absorptiometry) [40].

An analysis of DMFT/DMFT and body fat mass, measured by DXA, demonstrated a specific correlation with dietary habits (intake of sugar-sweetened beverages, frequency of sugar intake limited to main meals, frequency of food intake between meals) [41].

It is important to underline that even non-sweet snacks (e.g., chips/crisps, popcorn, and shrimp crackers) are potentially cariogenic [42] due to their content of extensively hydrolysed starch [43].

According to the pathogenesis model of caries based on the “ecological plaque hypothesis”, a greater role has been assigned to sugar intake in the aetiology. An increased frequency of fermentable sugar intake determined repeated conditions of low pH in biofilms, selecting an acid-tolerating bacterial community and cariogenic species [7]. Specifically, sucrose supplementation disrupted homeostasis and had the strongest cariogenic potential compared to glucose and lactose [44]. Therefore, dental caries are a consequence of an ecological shift in the balance of the beneficial oral microbiota, driven by a change in lifestyle and oral conditions. The caries risk is higher in individuals with impaired saliva flow and a sugar-rich diet, but it is reduced in those with appropriate oral hygiene and exposure to fluoride [7].

The goal of dental caries prevention is to preserve a strong tooth structure, prevent demineralization of enamel, and promote natural healing processes.

From the analysis of the caries protective factors related to oral hygiene, in the present study it emerged that children did not change their sleeping hygiene routine during the lockdown, continuing to brush their teeth before going to sleep (90.5% before vs. 88.6% during, *p* > 0.05). Moreover, 75% of them did not modify their tooth-brushing habits (Table 3).

Good oral hygiene habits related to tooth brushing before going to sleep and toothpaste choice were also maintained by comparing the period before lockdown and during lockdown (*p* > 0.05) (Table 3). Surprisingly, we observed that the use of fluoride toothpaste was carried out only “sometimes” (29.5% before lockdown, 29.5% during lockdown; *p* > 0.05), or was not used at all (15.5% before lockdown, 16.8% during lockdown; *p* > 0.05) by a high percentage of subjects. The percentage of children using non-fluoridated toothpaste was higher than that observed (6.4%) in a cross-sectional study conducted in New Zealand on 4723 children [45]. It is possible to highlight that almost half of the children in the study did not perform the correct topical fluor prophylaxis using fluoride toothpaste even before the lockdown. These data contrasted with the Italian guidelines for the promotion of oral health and the prevention of oral diseases, which underline the need to use fluoride [38]. They also contrasted with the American Academy of Pediatric Dentistry’s advice to brush teeth twice a day with a fluoridated toothpaste to provide continuing topical benefits [46]. We suppose that those results could be related to a social-vulnerability conditions (social disadvantage, scarce economic resources, poor education with lack of sensitivity to problems of dental prevention).

Fluoride is safe and highly effective to prevent children’s dental caries [46] and reduce caries, even when there is a greater intake of sugar in the diet [47].

Furthermore, 0.9% of the study subjects did not use toothpaste at all, and 32% did not have their own toothpaste but used their parents’. We cannot clarify to what extent these figures were influenced by the limited shopping possibilities during the lockdown.

Moreover, the data analysis related to eating frequency (e.g., snacks, biscuits, candies, chocolate) or drinking habits (e.g., milk, herbal teas—excluding water) before going to sleep and after brushing teeth highlighted a significant increase, from 0.77 ± 1.41 to 1.08 ± 1.70 times per week during the COVID-19 lockdown (*p* < 0.001). This erroneous habit was linked to ECC with the administration of bottles filled with fermentable liquids containing carbohydrates, rather than soothers dipped in honey or sugar [12,13]. These behaviours increased the cariogenic risk by nullifying an important moment of oral hygiene: the evening brushing. Regular tooth-brushing in the evening and the development of new dental caries presented a significant negative correlation [48]. In a recent Italian survey, it emerged that parents still were not fully trained and informed about the management of their child’s oral hygiene [49]. Thus, a parent/caregiver oral health promotion program is essential to control risk factors for their children’s oral health, as early as during the first months of life [31]. Even in the context of the COVID-19 pandemic [50], it is essential to focus on oral health education interventions at home to prevent carious pathology, which could expose the child to emergency dental treatments (e.g., acute pulpitis, acute apical periodontitis) [51].

According to the data of the study, 7.3% of the subjects had already been diagnosed with caries pathologies before the lockdown. During the lockdown, 10% experienced painful dental symptoms, and 2.7% experienced abscess pathologies.

Unfortunately, the COVID-19 lockdown initially limited routine oral care and prevention except for emergency and urgent interventions, but according to Brian and Weintraub, the pandemic offered an opportunity for the dental profession to shift more toward non-aerosolizing and prevention-focused approaches [52]. Attending periodic prevention appointments is essential for oral health and protective factors such as oral professional hygiene, application of high concentrations of fluoride gel, dental sealants, changes in diet, and reinforcement of child compliance [48].

Our results confirmed previous data on risk factors for early childhood caries [53].

As recently reported, applying a 25% AgNO3 solution followed by a commercially available 5% NaF containing functionalized tricalcium phosphate could be a solution for the prevention and control of dental caries [54].

The limitations of this study concern the poor sample size, as well as the lack of clinical data on oral health status and hygiene index.

## 5. Conclusions

This survey showed that during the COVID-19 lockdown, there was an increased risk factor of caries compared to protective factors. This was due to an increased intake of fermentable sugars, frequent consumption of dietary sugars, frequent intake of snacks between meals, incorrect oral-habit hygiene, and inadequate topical fluor prophylaxis caused by an occasional use of fluoride toothpaste. It was concluded that in this pandemic period, a caries-prevention program aimed at parents and children is essential to intercept caries pathology risk factors and encourage protective behaviors, while also considering the prolongation of the emergency period.

## Figures and Tables

**Table 1:** Population characteristics and anthropometrics.

	Whole Samples (*n* = 220)	*p*
Age	9.10 ± 2.86	<0.001
Gender (F)	111 (50.5)	<0.001
Weight (kg)	35.84 ± 15.72	<0.001
Height (cm)	137.01 ± 18.48	<0.05
BMI (kg/cm^2^)	18.45 ± 17.74	<0.001
TMI (kg/cm^2^)	13.63 ± 3.65	<0.001
**Type of lactation (First 6 months)**		
Formula milk	43 (19.5)	
Mixed lactation	70 (31.8)	
Breastfeeding	107 (48.6)	

Values are expressed as mean ± SD for continuous variables or as number and percentage (*n* (%)) for categorical variables. Female: F; Body Mass Index: BMI; Triponderal Mass Index: TMI. The Shapiro–Wilk test was performed to evaluate variables distribution. Variables were considered non-normally distributed for *p* < 0.05.

**Table 2:** Eating habits during lockdown.

	*n* = 220
**KIDMED TEST**	
Had a fruit every day	145 (65.9)
Had a second fruit every day	72 (32.7)
Had fresh or cooked vegetables regularly once a day	135 (61.4)
Had fresh or cooked vegetables more than once a day	63 (28.6)
Consumed fish regularly (at least 2–3 times per week)	110 (50.0)
Went more than once a week to a fast-food (hamburger) restaurant	20 (9.1)
Liked pulses and ate them more than once a week	111 (50.5)
Consumed pasta or rice almost every day (5 or more times per week)	197 (89.5)
Had cereals or grains (bread, etc.) for breakfast	131 (59.5)
Consumed nuts regularly (at least 2–3 times per week)	61 (27.7)
Used olive oil at home	219 (99.5)
Skipped breakfast	56 (25.5)
Had a dairy product for breakfast (yogurt, milk, etc.)	191 (86.8)
Had commercially baked goods or pastries for breakfast	165 (75.0)
Had two yogurts and/or some cheese (40 g) daily	65 (29.5)
Had sweets and candy several times every day	110 (50.0)
**KIDMED score**	5.00 [3.00]
Low adherence	50 (22.7)
Medium adherence	129 (58.6)
High adherence	41 (18.6)
**Eggs**	2.00 [1.00]
**Water**	
<1 Liters	103 (46.8)
1–2 L Liters	102 (46.4)
>2 L Liters	15 (6.8)
**Eating-habit changes**	
No change	112 (50.9)
More sweets	45 (20.5)
Less sweets	15 (6.8)
Compensatory food and sedentary for boredom	44 (20.0)
More sugary beverages	4 (1.8)
**Increase of sweets consumption (yes)**	113 (51.4)

Values are expressed as number and percentage (*n* (%)) for categorical variables. Mediterranean Diet Quality Index: KIDMED.

**Table 3:** Tooth-brushing habits before and during the lockdown.

	before Lockdown (*n* = 220)	during Lockdown (*n* = 220)	*p*
**Use of fluoride toothpaste**			
Never	34 (15.5)	37 (16.8)	0.025
Sometimes	65 (29.5)	65 (29.5)	
Often	50 (22.7)	50 (22.7)	
Always	71 (32.3)	60 (30.9)	
**Teeth brushing before going to sleep (yes)**	199 (90.5)	195 (88.6)	0.388
**Drinking/eating after brushing teeth (times/week)**	0.77 ± 1.41	1.08 ± 1.70	<0.001
**Dental pain or discomfort (yes)**		22 (10.0)	
**Dental abscesses (yes)**		6 (2.7)	
**Toothpaste used**			
Personal		146 (66.4)	
Parents’ toothpaste		72 (32.7)	
None		2 (0.9)	
**Tooth-brushing habits**			
Not changed		165 (75.0)	
Less frequency brushing		33 (15.0)	
More frequency brushing		22 (10.0)	

Values are expressed as mean ± SD for continuous variables or as number and percentage (n (%)) for categorical variables. The Mann–Whitney and the McNemar’s analysis were used to investigate the difference between continuous and categorical variables before and during the lockdown. Results were significant for *p*-value < 0.05.

## Appendix A

**Table A1:** Web Survey.

	Questions	Answers
	1. Parent 1 gender	Male/Female
	2. Parent 1 weight	Weight in kg
	3. Parent 1 height	Height in cm
	4. Parent 1 ethnicity	Caucasian/Hispanic/Afro-American/Asian
	5. Parent 1 nationality is Italian?	Yes/No
	6. Parent 1 degree of education	None/Elementary School Diploma/Superior School Diploma/Master’s Degree/Post-Master’s Degree Diploma
**Socio-economic** **status of the family**	7. Parent 1 occupation	Employee/Manager/Teacher/Freelance Merchant/Artisan/Parasanitary personnel/Workmen/Retired/Unemployed/ Armed forces and security/Other
	8. Parent 2 gender	Male/Female
	9. Parent 2 weight	Weight in kg
	10. Parent 2 height	Height in cm.
	11. Parent 2 ethnicity	Caucasian/Hispanic/Afro-American/Asian
	12. Parent 2 nationality: Italian?	Yes/No
	13. Parent 2 degree of education	None/Elementary School Diploma/Superior School Diploma/Master’s Degree/Post-Master’s Degree Diploma
	14. Parent 2 occupation	Employee/Manager/Teacher/Freelance/ Merchant/Artisan/Parasanitary personnel/Workmen/Retired/Unemployed/ Armed forces and security/Other
	15. Child gender	Male/Female
	16. Age	4/5/6/7/8/9/10/11/12/13/14
	17. Region of residence	Valle D’Aosta/Piemonte/Liguria/Lombardia Trentino-Alto Adige/Veneto/Friuli-Venezia Giulia/Emilia Romagna/Toscana/Umbria Marche/Lazio/Abruzzo/Molise/Campania Puglia/Basilicata/Calabria/Sicilia/Sardegna
**Personal and anthropometric data for the child**	18. Province of residence	AG/AL/AN/AO/AQ/AR/AP/AT/AV/BA/BT/BL BN/BG/BI/BO/BZ/BS/BR/CA/CB/CL/CI/CE CT/CZ/CH/CO/CS/CR/KR/CN/EN/FM/FE/FI FG/FC/FR/GE/GO/GR/IM/IS/SP/LT/LE/LC LI/LO/LU/MC/MN/MS/MT/VS/ME/MI/MO MB/NA/NO/NU/OG/OT/OR/PD/PA/PR/PV PG/PU/PE/PC/PI/PT/PN/PZ/PO/RG/RA/RC RE/RI/RN/RM/RO/SA/SS/SV/SI/SR/SO/TA TE/TR/TO/TP/TN/TV/TS/UD/VA/VE/VB/VC VR/VV/VI/VT
	19. Child height	Height in cm
	20. Child weight	Weight in kg
	21. Lactation prevalent in the first 6 months of life	Breastfeeding/Formula milk/Mixed
	22. Before the lockdown, did the child have caries?	Yes/No
	23. During the lockdown, has the child experienced teeth pain?	Yes/No
	24. During the lockdown, did the child have dental abscesses?	Yes/No
	25. Before the lockdown, how many times a day did the child brush their teeth?	0/1/2/3/4/5
**Child oral health before and during lockdown**	26. Before the lockdown, did the child use fluoride toothpaste?	Never/Sometimes/Often/Always
	27. During the lockdown, did the child use fluoride toothpaste?	Never/Sometimes/Often/Always
	28. During the lockdown, has the child used the parents’ toothpaste or personal toothpaste, or none?	No toothpaste/His/her personal toothpaste/Parents’ toothpaste
	29. During the lockdown, has the child’s teeth-brushing habits changed?	Have not changed/The frequency of tooth cleaning has increased/The frequency of tooth cleaning has decreased
	30. Before the lockdown, did the child brush their teeth before going to sleep?	Yes/No
	31. During the lockdown, has the child brushed their teeth before going to sleep?	Yes/No
	32. Before the lockdown, how many meals did the child have during the day?	0/1/2/3/4/5/6/7/8
	33. During the lockdown, how many meals has the child eaten a day?	0/1/2/3/4/5/6/7/8
	34. Before the lockdown, did the child eat between meals?	Yes/No
**Eating habits of the child before and during lockdown**	35. During the lockdown, has the child eaten between meals?	Yes/No
	36. Before the lockdown, did the child eat candies/chocolate, or drink milk/herbal tea before going to sleep?	Never/1 time a week/2 times a week/3 times a week/4 times a week/5 times a week/6 times a week/7 times a week
	37. During the lockdown, has the child eaten snacks, cookies, or drank milk before going to sleep?	Never/1 time a week/2 times a week/3 times a week/4 times a week/5 times a week/6 times a week/7 times a week
	38. Did the child take probiotic supplements before the lockdown?	Yes/No
	39. Has the child taken probiotic supplements during the lockdown?	Yes/No
	40. During the lockdown, how did the diet change—increase or decrease in terms of sugary food?	Not modified/Preferred sweets more/Reduced intake of sweet foods/Drank more sugary drinks/Reduced intake of sugary drinks/He/she was bored and sedentary and eating activities compensated
	41. During the lockdown, has the child consumed a fruit or fruit centrifuge/juice once a day?	Yes/No
	42. During the lockdown, has a second fruit been consumed every day by the child?	Yes/No
	43. During the lockdown, has the child regularly consumed vegetables once a day?	Yes/No
	44. During the lockdown, did the child eat multiple servings of raw or cooked vegetables per day?	Yes/No
	45. During the lockdown, has fish been consumed regularly, twice or three times a week, by the child?	Yes/No
	46. During the lockdown, has fast food been consumed more than once a week by the child?	Yes/No
	47. During the lockdown, have legumes been consumed more than once a week by the child?	Yes/No
	48. During the lockdown, has pasta or rice been consumed every day (5 or more days a week) by the child?	Yes/No
**Child feeding during lockdown**	49. During the lockdown, has the child consumed cereals (cornflakes) or bread for breakfast?	Yes/No
	50. During the lockdown, have dry fruits like almonds or nuts been consumed regularly (2–3 times a week) by the child?	Yes/No
	51. During the lockdown, has the child consumed olive oil?	Yes/No
	52. During the lockdown, has the child skipped breakfast?	Yes/No
	53. During the lockdown, has the child eaten dairy products such as yogurt or milk for breakfast?	Yes/No
	54. During the lockdown, has the child consumed cookies, cakes, or snacks for breakfast?	Yes/No
	55. During the lockdown, has the child consumed 2 yogurts or cheese every day?	Yes/No
	56. During the lockdown, has the child eaten candies, sweetened foods (cookies, cakes, ice creams etc.) more often?	Yes/No
	57. During the lockdown, has the child consumed more sweetened foods?	Yes/No
	58. During the lockdown, how many eggs did the child consume per week?	0/0.5/1/2/3/4/5/6/7/8/9/10
	59. During the lockdown how much water did the child drink?	Less than 1 L/Between 1 L and 2 L/More than 2 L
	60. Before the lockdown, what was the lifestyle habit of the child?	Sedentary hypokinetic/Sedentary/Moderately Active/Intense physical activity
	61. During the lockdown, what was the lifestyle habit of the child?	Sedentary hypokinetic/Sedentary/Moderately active/Intense physical activity
	62. Before the lockdown, how many hours of sleep did the child get?	Less than 7 h/Between 7 and 9 h/More than 9 h
	63. During the lockdown, how many hours of sleep did the child get?	Less than 7 h/Between 7 and 9 h/More than 9 h
	64. Before the lockdown, did the child do any physical activities?	Yes/No
**Child lifestyle before and during lockdown**	65. Before the lockdown, what sport did the child play?	Fitness Course/Running/Walking/Cycling, Football/Basket/Volleyball/Martial Arts/Tennis, Paddle/Swim/Dance/Artistic Gymnastics
	66. Before the lockdown, how many weekly hours of physical activity did the child do?	1/2/3/4/5/6/7/8/9/10/11/12/13/14/15/16/17/18/19/20
	67. During the lockdown, did the child play any sports?	Yes/No
	68. What sport did the child play during lockdown?	Fitness Course/Running/Walking/Cycling, Football/Basket/Volleyball/Martial Arts/Tennis, Paddle/Swim/Dance/Artistic Gymnastics
	69. During the lockdown, how many weekly hours of physical activity did the child do?	1/2/3/4/5/6/7/8/9/10/11/12/13/14/15/16/17/18/19/20
	70. Before the lockdown, how many hours a day did the child spend playing videogames or watching TV?	0/0.5/1/2/3/4/5/6/7/8
	71. During the lockdown, how many hours per day did the child spend playing videogames or watching tv?	0/0.5/1/2/3/4/5/6/7/8
	72. During the lockdown, how many hours per day did the child spend doing online classes?	0/0.5/1/2/3/4/5/6

AG, Agrigento; AL, Alessandria; AN, Ancona; AO, Aosta; AQ, L’Aquila; AR, Arezzo; AP, Ascoli Piceno; AT, Asti; AV, Avellino; BA, Bari; BT, Barlettta-Andria-Trani; BL, Belluno; BN, Benevento; BG, Bergamo; BI, Biella; BO, Bologna; BZ, Bolzano; BS, Brescia; BR, Brindisi; CA, Cagliari; CB, Campobasso; CL, Caltanissetta; CI, Carbonia-Iglesias; CE, Caserta; CT, Catania; CZ, Catanzaro; CH, Chieti; CO, Como; CS, Cosenza; CR, Cremona; KR, Crotone; CN, Cuneo; EN, Enna; FM, Fermo; FE, Ferrara; FI, Firenze; FG, Foggia; FC, Forlì-Cesena; FR, Frosinone; GE, Genova; GO, Gorizia; GR, Grosseto; IM, Imola; IS, Isernia; SP, La Spezia; LT, Latina, LE, Lecce; LC, Lecco; LI, Livorno; LO, Lodi; LU, Lucca; MC, Macerata; MN, Mantova; MS, Massa-Carrara; MT, Matera; ME, Messina; MI, Milano; MO, Modena; MB, Monza e Brianza; NA, Napoli; NO, Novara; NU, Nuoro; OG, Ogliastra; OT, Olbia-Tempio; OR, Oristano; PD, Padova; PA, Palermo; PR, Parma; PV, Pavia; PG, Perugia; PU, Pesaro e Urbino; PE, Pescara; PC, Piacenza; PI, Pisa; PT, Pistoia; PN, Pordenone; PZ, Potenza; PO, Prato; RG, Ragusa; RA, Ravenna; RC, Reggio Calabria; RE, Reggio Emilia; RI, Rieti; RN, Rimini; RM, Roma; RO, Rovigo; SA, Salerno; SS, Sassari; SV, Savona; SI, Siena; SR, Siracusa; SO, Sondrio; TA, Taranto; TE, Teramo; TR, Terni; TO, Torino; TP, Trapani; TN, Trento; TV, Treviso; TS, Trieste; UD, Udine; VA, Varese; VE, Venezia; VB, Verbano-Cusio-Ossola; VC, Verbelli; VR, Verona; VS, Medio Campidano; VV, Vibo Valentia; VI, Vicenza; VT, Viterbo.
